# Beyond development: LINX/Islr2 expression and TrkA interaction in the adult nervous system under neuroinflammatory challenge

**DOI:** 10.3389/fnmol.2026.1863350

**Published:** 2026-09-23

**Authors:** Pamela Rosso, Elena Fico, Viviana Triaca, Annamaria Di Criscio, Ari-Pekka Koivisto, Katja Kuokkanen, Mikko Ares, Kaisa Auvinen, Andrii Domanskyii, Johanna Kela, Carina Stenfors, Paola Tirassa

**Affiliations:** 1 Institute of Biochemistry and Cell Biology, National Research Council (CNR), International Campus A. Buzzati-Traverso, Rome, Italy; 2 Department of Sense Organs, Sapienza University of Rome, Rome, Italy; 3 Orion Corporation Orion Pharma, R&D, Espoo, Finland

**Keywords:** dorsal root ganglion, LPS, neurodegeneration, neurons, neurotrophins

## Abstract

**Introduction:**

LINX, also known as immunoglobulin superfamily containing leucine-rich repeat 2 (Islr2), was identified as a TrkA pathway component and demonstrated to modulate the NGF/TrkA-mediated axonal growth, guidance, and branching during development. However, there is limited information on LINX expression in the adult nervous system, and the possible functions of LINX in mature neurons remain unexplored. These two questions are addressed by the present study, which first investigates LINX expression in the adult spinal cord (SC), dorsal root ganglia (DRG), and cerebral cortex (Cx), and subsequently explores LINX/TrkA functions using the DRG organ culture model.

**Methods:**

Different commercially available anti-LINX antibodies were tested, and the most selective antibody was used to investigate LINX expression in the SC, Cx, and DRG of healthy rats, and to explore the LINX/TrkA action in DRG organ cultures using a model of neuroinflammation/injury induced by lipopolysaccharides (LPS). The LINX/TrkA interaction was demonstrated by immunoprecipitation in the Cx of healthy and LPS-treated rats. The DRG from healthy and LPS-treated rats were dissected and cultured in the presence of anti-LINX, with or without stimulation using NGF, and a TrkA selective inhibitor. TrkA phosphorylation levels and the expression of the axonal growth marker GAP43 were analyzed by bio-molecular assays and confocal immunofluorescence microscopy.

**Results:**

The WB analysis shows that LINX is expressed in the adult central and peripheral nervous systems, and the immunoprecipitation analysis demonstrates LINX and TrkA interaction in the Cx of both adult healthy and LPS-treated rats. *In vitro* experiments using an LPS-DRG model confirm the association between LINX and TrkA. However, it was found that while anti-LINX administration abolishes the effects of NGF and TrkA activation in LPS-DRG, it results in GAP43 increase in the presence of a TrkA inhibitor.

**Discussion:**

Our study confirms that LINX is expressed in the adult central nervous system and demonstrates that the LINX/TrkA interaction is maintained following LPS-induced systemic inflammation. Moreover, our findings that antibody-mediated perturbation of endogenous LINX is associated with the modulation of axonal growth markers in adult LP-DRG cultures, in serum-free conditions and/or in the presence of a TrkA inhibitor, suggest a potential role for LINX in regulating neurotrophin-dependent responses in injured adult neurons.

## 1 Introduction

LINX, also known as immunoglobulin superfamily containing leucine-rich repeat 2 (Islr2) and KIAA1465 protein, is a type I transmembrane protein and a member of the LIG family of proteins characterized by five tandem leucine-rich repeats (LRRs), and an immunoglobulin (Ig)-like domain (Abudureyimu et al., 2018). LINX was found in the spinal cord (SC), dorsal root ganglia (DRG), sympathetic ganglia, and brain of embryos and developing animals, and in rodent adult cortex (Cx) and SC (Abudureyimu et al., 2018; Mandai et al., 2009). As an LRR transmembrane protein, LINX is identified as a functional modulator and co-receptor component for tropomyosin receptor kinase A (TrkA) and other receptor tyrosine kinases, such as Ret. In fact, axonal projection defects found in LINX null mice resemble those in mice lacking nerve growth factor (NGF), TrkA, and Ret (Mandai et al., 2009; Mandai et al., 2014; Panza et al., 2015). However, in contrast to NGF/TrkA mutants, LINX depletion has little impact on the control of neuronal survival, indicating a specific role of LINX in the modulation of Trk-mediated axon growth, guidance, and branching during development (Mandai et al., 2009). No information about the actions of LINX in the adult nervous system is currently available, although the presence of LINX in the adult mouse brain was shown in a previous study (Abudureyimu et al., 2018). Furthermore, whether or how LINX might be involved in adult axon/nerve maintenance and/or repair has not yet been investigated. As largely reported, NGF is required for neuronal survival, differentiation, and activities throughout the life span (Sofroniew et al., 2001), and the alteration of the NGF/TrkA pathway characterizes the mechanism of neuronal injury and supports the neuroprotective and regenerative roles of TrkA ligands (Triaca et al., 2020). It is therefore possible to hypothesize that, being associated with TrkA, LINX expression in adult nervous structures might be affected and/or participate in the mechanism(s) regulating axon growth in physiological and/or pathological conditions. To explore this hypothesis, we have investigated the LINX expression in the SC, DRG, and Cx of adult healthy rats and in a model of neuroinflammation/injury induced by intraperitoneal injection of a subtoxic dose of lipopolysaccharides (LPS). The challenge with LPS is one of the most commonly used models to study NGF/TrkA in regulating immune cell responses (Prencipe et al., 2014), activating microglia (Fodelianaki et al., 2019), and, more recently, exploring the effects of inflammation on NGF processing (Makoudjou et al., 2024). In the DRG *ex vivo* (organotypic cultures) or primary neuronal culture, LPS induces neuroinflammation and the release of cytokines, and neuropeptides, as well as peripheral sensitization (Nürnberger et al., 2022). Furthermore, it is often used as an alternative approach to study the mechanisms of inflammatory pain, which involve NGF and its receptors (Barker et al., 2020). The *ex vivo* DRG organ culture is also suggested to mimic axotomy-induced changes in neurotrophic factors and other growth-regulatory molecules that prime neurons for regeneration (Krishnan et al., 2021). In addition, the LPS challenge has proved to compromise the blood–brain barrier in the brain and DRG (Reinhold and Rittner, 2020), increase paracellular permeability (Bein et al., 2017), and stimulate endocytic internalization and transient membrane destabilization (Zanoni et al., 2011). This, in turn, allows for the aberrant penetration of macromolecules into the organs. Thus, organotypic cultures of adult DRG derived from healthy or LPS-challenged rats were also used as a tool to explore the antibody-mediated perturbation of endogenous LINX on the regulation of axon growth and its association with TrkA activation.

## 2 Materials and methods

### 2.1 Animals

CD1 male mice from the in-house colony and adult male, pathogen-free, Sprague–Dawley (SD) rats (body weight, 200–250 g), purchased by Envigo (Italy) were used in this study. From the moment of their arrival, the rats were left undisturbed for 2 weeks in their cages to recover from the stress of transport and to be habituated to the new animal facility conditions. A veterinarian also checked their health status before starting the experiments. Both the CD1 mice and the rats were maintained in a 12-h light–dark cycle and were provided with food and water *ad libitum* throughout the experimental period. All procedures involving animal care or treatments were performed according to the intramural Committee and Institutional Guidelines of the Italian National Research Council, in compliance with the National (D. Lgs 26/2014) and European Union legislation guidelines for animal welfare (2010/63/EU), and approved by the Italian Ministry of Health (Authorization n° 941/2024-PR prot. 82945.83). All efforts were taken to limit the number of animals in the study, and the use of the DRG culture method (paragraph 2.4) was selected to align with the 3Rs principles, especially reduction and refinement. The choice of an organoid culture to investigate the second part of this study aligns with the purpose of New Approach Methodologies (NAMs) without being explicitly listed as one of them.

### 2.2 LINX antibody screening in mouse and rat nervous system tissues

Since LINX expression in the central nervous system has been previously reported during the late embryonic/early post-natal period in mice by using anti LINX/Islr2 antibody by R&D Systems (AF4526) and Abnova (PAB15698), these antibodies were preliminarily used to set up the Western Blot (WB) conditions and to analyze the expression in the entire brain of adult male rats (Rat Cx), as well as in one postnatal day (Br P1) and 6 months (Br 6 Ms) old CD1 male mice. The brains were removed under anesthesia; five PD1 mice were euthanized by decapitation, and their brain tissues were pooled. Additionally, the total brain (without the cerebellum; Br), the spinal cord (SC), and the olfactory bulbs (OB) were surgically removed from 6 Ms. CD1 male mice (*n* = 4). Following this preliminary analysis, the Cx, SC, and DRG were dissected from five adult male rats, and the extracted total proteins were used to further verify LINX expression using other commercially available antibodies, such as anti-Islr2 from Abnova (PAB15698), Sigma-Aldrich (AV49579), Biorbyt (orb46760), and ProSci (ProSci Cat n.26–422) in WB analysis, as described below.

### 2.3 LPS model

A total of 20 adult male rats were randomly divided into two experimental groups: the untreated group, named control (CTR *n* = 6), and lipopolysaccharides (LPS *n* = 14) group, which received a single intraperitoneal injection of lipopolysaccharides (LPS, 5 mg/kg b.w.). For biomolecular and *in vitro* investigations, the rats from both groups were euthanized by decapitation after being sedated with tiletamine/zolazepam (40 mg/kg) and xylazine (5/10 mg/kg) intraperitoneally. The Cx, SC, and DRG were dissected and washed in saline. The Cx, SC, and a small part of DRG (*n* = 4 per rat) were coded and stored in a tube at −80 °C until used, while the remainder of the collected DRG were used in the *in vitro* experiments described below.

### 2.4 Dorsal root ganglion (DRG) organotypic culture protocol

The DRG from CTRL- and LPS-treated rats were dissected, as described by Sleigh et al. (2016). Briefly, after shaving the fur and disinfecting with alcohol, the spinal column was isolated and removed and placed on a refrigerated dissection plate, maintaining the rostral to caudal direction, cleared form soft tissues and nerve, and cut longitudinally to expose the SC and the DRG. The SC was removed from the vertebral canal, the lumbar DRG were dissected under a stereoscopic magnifier and divided into two pool groups, CTRL and LPS, to have enough DRG to be treated and processed for different analyses according to the experimental plan. After washing 2–3 times with sterilized PBS, the DRG from the CTRL pool (approximately 60 DRGs) or LPS pool (approximately 160 DRGs) were randomly transferred to single wells (n = 5/6 per well) in new sterile culture dish and filled with approximately one-third of the culture Roswell Park Memorial Institute (RPMI 1640) medium containing antibiotic-antimycotic solution for 30′-1 h into the incubator at 37 °C and 5% CO2. To characterize the LINX/TrkA interaction and evaluate the effects of anti-LINX antibody (Abnova, PAB15698; 20 μg/mL) treatment, each well with DRG explants from CTRL and LPS rats was incubated with anti-LINX, with or without stimulation with NGF (100 ng/mL), and anti-LINX plus the TrkA selective inhibitor GW441756 (15 μM). The experiment included the following treatment groups: CTRL; LPS; LPS + NGF; LPS + *α*-LINX; LPS + TrkA Inhibitor; LPS + α-LINX + TrkA Inhibitor; LPS + NGF + α-LINX. Purified Normal Rabbit IgG from ProSci Incorporated (Poway, CA, United States) was used as a control (20 μg/mL) in all experiments. For each treatment group, three technical replicate wells were prepared from the same CTR and LPS DRG pool. At 48 h after treatment, the DRG were collected for protein extraction and processed for biomolecular analysis or for IF.

### 2.5 Protein quantification and immunoblotting

Rat and mouse tissues, as well as from cultured DRG, were extracted and homogenized by ultrasonication in RIPA buffer (50 mM Tris–HCl, pH 7.4; 150 mM NaCl; 5 mM EDTA; 1% Triton X-100; 0.1% SDS; 0.5% sodium deoxycholate; 1 mM PMSF; 1 mg/mL leupeptin), and kept in a cold room on a rotary shaker for 2 h to allow the complete tissue disaggregation and cell lysis, and then centrifuged at 10,000 g for 30 min at 4 °C. The supernatants were collected, protein concentration was measured by Bio-Rad assay, and then stored in aliquots at −80 °C until use. For samples shown in Figures 1–3 (Results section), protein extracts were prepared from three independent biological replicates, each obtained from a different animal (*n* = 3). Sample protein extracts (20 μg of total protein) were dissolved in loading buffer (0.1 mol/L of Tris–HCl buffer, pH 6.8, containing 0.2 mol/L of DTT, 4% sodium dodecyl sulfate, 20% glycerol, and 0.1% bromophenol blue), separated by sodium dodecyl sulfate polyacrylamide gel electrophoresis or Bolt 4 to 12%, Bis-Tris precast gels, and electrophoretically transferred to nitrocellulose membranes (0.45 mm). The membranes were incubated for 1 h at RT with 5% non-fat-dried milk dissolved in tris-buffered saline with tween-20 (TBS-T; 10 mmol/L of Tris, pH 7.5, 100 mmol/L of NaCl, and 0.1% Tween-20), washed three times for 10 min each in TBS-T, and then incubated overnight at 4 °C with primary antibodies anti-LINX (1:1000 for rat tissues, 1:500 for mouse tissue): anti-LINX from Abnova (PAB15698), anti-LINX from ProSci (Cat. N 26–422), anti-LINX from Sigma Aldrich (AV49579), and anti-LINX from Biorbyt-HRP (orb467608). After incubation, membranes were washed three times for 10 min each in TBS-T and incubated for 1 h at RT with anti-rabbit IgG (1:5000–1:10000; Cell Signaling Technology, Danvers, MA, and 1:2000, Perkin Elmer) and anti-sheep IgG-HRP (1:2000, TrueBlot, Rockland) secondary antibodies. The blots were developed with Clarity Western ECL Substrate (Bio-Rad) or ECL substrate (Pierce) kits as the chromophores. β-actin-HRP (1:5000, Santa Cruz Biotechnology; 1:20000, Sigma) was chosen as housekeeping protein.

**Figure 1:**
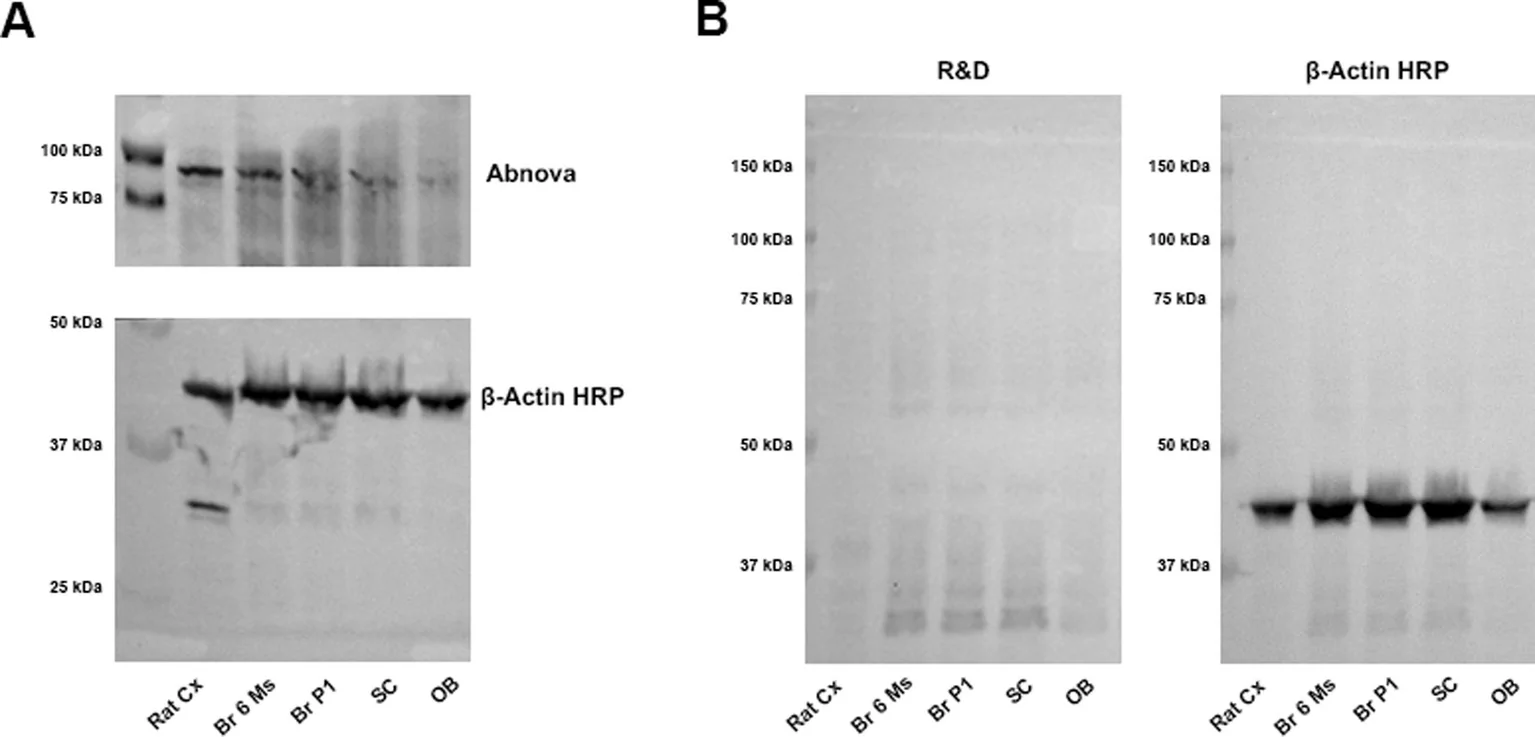
LINX expression in adult mice and rats. **(A)** Representative western blotting (WB) of extracts from young adult rat cortex (rat Cx) and CD1 male mice brain 6 months (Br 6 Ms), CD1 male mice brain from postnatal day 1 (Br P1), CD1 male mice 6 months spinal cord (SC) and CD1 male mice 6 months olfactory bulbs (OB) using Abnova and R&D anti-LINX antibodies. **(B)** β-actin was used as a loading control.

**Figure 2:**
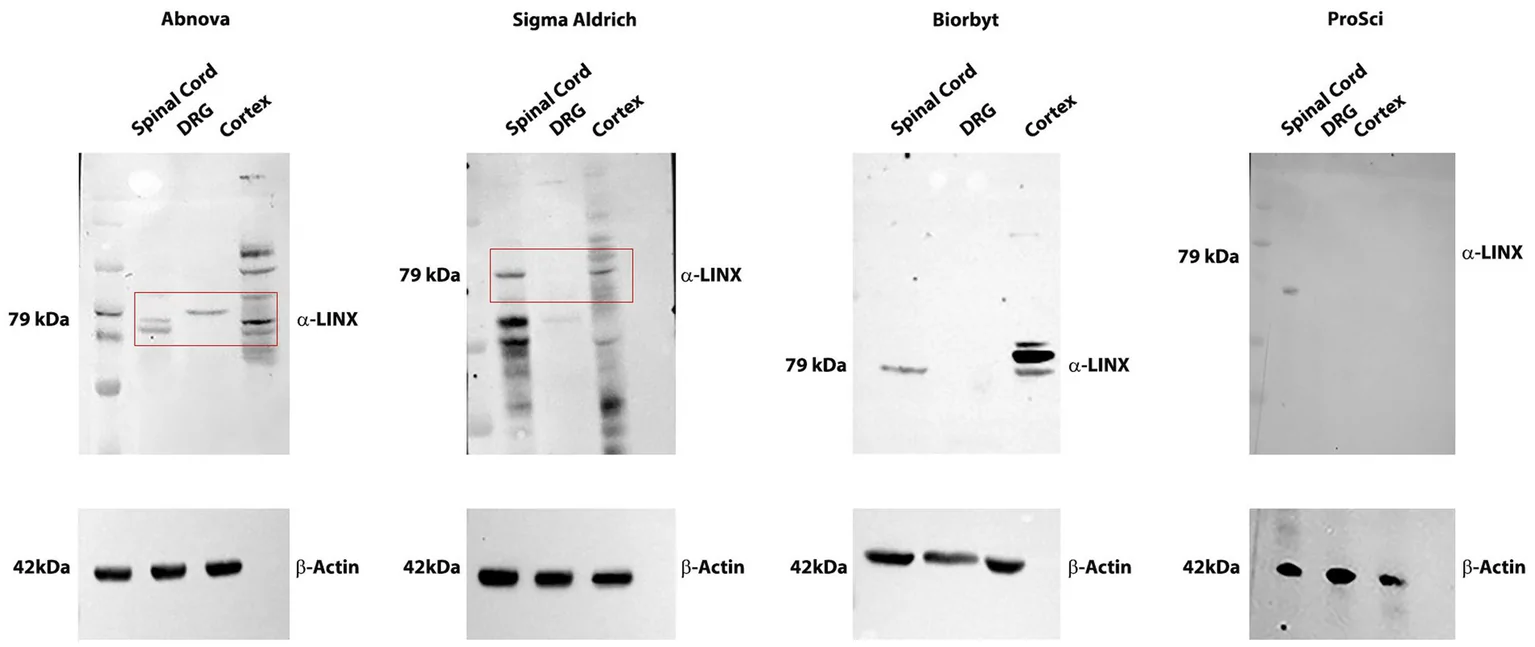
LINX expression in adult rats using different commercial anti-LINX antibodies. Representative western blotting (WB) of extracts from spinal cord, dorsal root ganglia (DRG), and cortex (Cx) of adult male rat brain, using four different commercial anti-LINX antibodies: Abnova, Sigma-Aldrich, Biorbyt, and ProSci. β-actin was used as a loading control.

**Figure 3:**
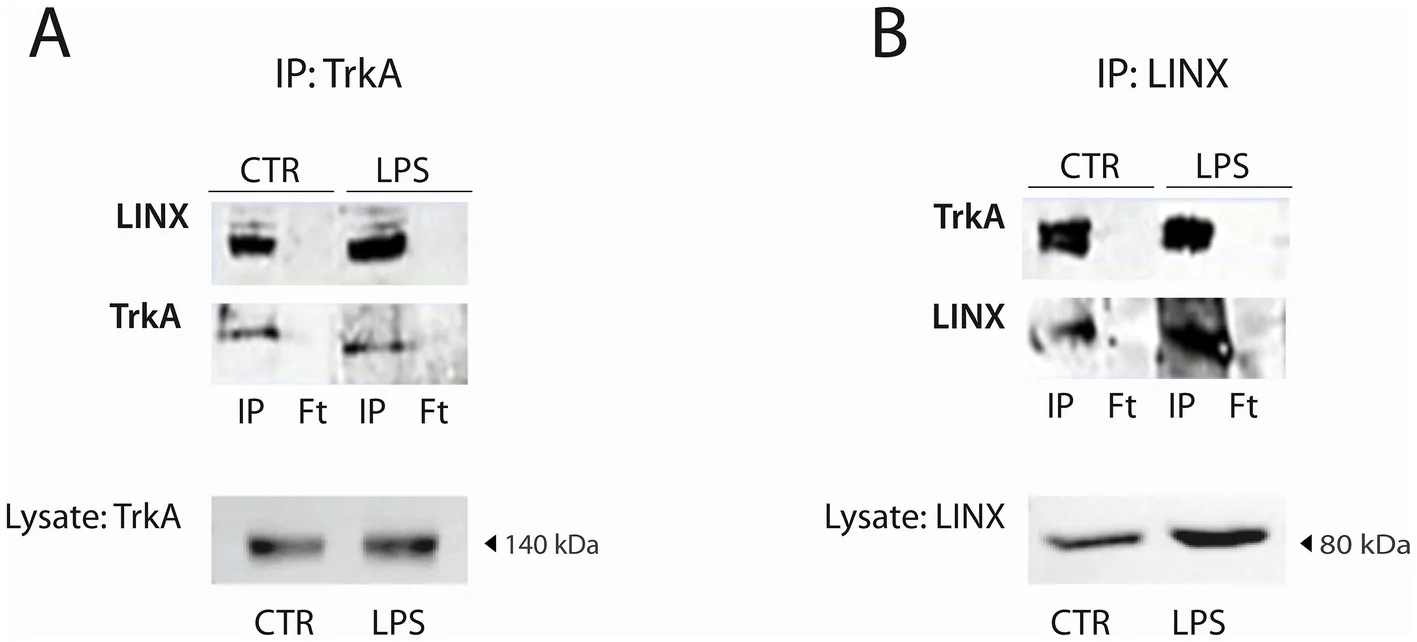
LINX-TrkA immunoprecipitation. Representative LINX and TrkA immunoprecipitation (IP) experiments show LINX-TrkA binding in cortical extracts of both untreated (CTRL) and LPS-treated (LPS) rats. **(A)** TrkA IP was performed with anti-TrkA-specific antibody (4 μL) in cortical extracts from CTRL or LPS-challenged rats, and LINX was probed with HRP-conjugated anti-LINX antibodies. **(B)** In the reciprocal experiment, LINX was immune-precipitated by rabbit anti-LINX antibodies (5 μg), and TrkA was probed by HRP-conjugated TrkA-specific antibody. Lysates were probed for both TrkA and LINX, as also shown in Supplementary Figure S3C.

### 2.6 PathScan Sandwich ELISA Kits

Two different PathScan Sandwich ELISA Kits were used to detect the phosphorylation of neurotrophin-specific receptors TrkA (Tyr490) and TrkA (Tyr674/Tyr675) in Dorsal Root Ganglia (DRG) protein lysates from organotypic cultures: PathScan Phospho-TrkA (Tyr490) Sandwich ELISA Kit (#7210), PathScan Phospho-TrkA (Tyr674/675) Sandwich ELISA Kit (#7212). The required number of microwells, coated with the specific primary Anti-TrkA antibody, was broken and placed into the strip holder at room temperature (RT) to be ready to be used. One hundred microliters of diluted DRG lysates were added to the appropriate well. The plate was incubated for 2 h at 37 °C. The wells were washed four times with 1X wash buffer. A measure of 100 μL of reconstituted detection antibody (Phospho-TrkA – Tyr490 – Rabbit Detection Antibody; Phospho TrkA – Tyr674/675 – Rabbit Detection Antibody, depending on the kit used) was added to each well. The plate was incubated at 37 °C for 1 h, and the wash procedure was repeated. One hundred μl of reconstituted HRP-linked secondary antibody (anti-rabbit IgG, HRP-linked antibody, ELISA formulated) was added to each well, and the plate was incubated for 30 min at 37 °C. Then, the wash procedure was repeated, and 100 μL of TMB substrate was added to each well and incubated for 10 min at 37 °C. At the end, 100 μL of STOP solution was added to each well, and the plate was gently shaken for a few seconds. The reaction started, and the initial blue color of the positive reaction changed to yellow upon addition of STOP solution. The absorbance of the reaction was read at 450 nm using a spectrophotometer within 30 min after adding STOP Solution.

### 2.7 Immunoprecipitation (IP) studies

A total of 2 mg of rat cortical protein extract was added to Dynabeads-Protein G and eluted with 0.1 M citrate buffer (pH 2.3) according to the manufacturer’s instructions (Invitrogen). Immunoprecipitation (IP) of LINX was performed using rabbit anti-LINX antibodies from Sigma and Abnova (5 μg/50 μL beads). Normal IgG was used as an isotype control in IP (Santa Cruz, sc-2027).

LINX IP was probed with rabbit anti-TrkA-HRP (Bioss) or anti-TrkA (sc-118; Santa Cruz Biotechnology) followed by the corresponding HRP secondary antibody (TrueBlot, Rockland, United States, 1:1000, 1 h, RT). TrueBlot secondary antibodies were used to detect only the native, non-reduced form of IgG and provide increased specificity and low background from contaminating IgG. In the reciprocal experiment, TrkA was immunoprecipitated in the cortical extracts by using the rabbit anti-TrkA-specific antibody (a generous gift from Prof. L. Reichardt; 4 μL/50 μL beads), and LINX was probed with rabbit anti-LINX-HRP antibody (Biorbyt). The blots were then stripped for 20′ with Restore WB stripping buffer (Thermo Fisher), washed in TBS, blocked with 7% non-fat dried milk (1 h, RT), and re-probed with respectively anti-LINX-HRP or anti-TrkA-HRP antibody (Bioss).

### 2.8 Immunofluorescence whole mount staining of DRG

The DRGs were fixed with 4% paraformaldehyde for 24 h. After fixation, the samples were washed in 0.1 M PBS for 24 h, permeabilized with 1% Triton X-100 for 24 h, and washed again in PBS. Furthermore, the samples were treated with 10% normal donkey serum (Invitrogen), containing 1% Triton X-100, for 24 h to block non-specific antibody binding, washed in PBS containing 1% Triton X-100, and incubated in a mix solution of anti-GAP43 (1:100, Santa Cruz Biotechnology, sc-10786) and anti-TrkA (1:100, sc-118, Santa Cruz Biotechnology) for 24 h. Next, the samples were washed in PBS and incubated in a mix solution of secondary anti-rabbit-Alexa 488 and anti-mouse-Alexa 546 (1:1000, Invitrogen) for 24 h. Finally, the samples were placed in PBS containing DAPI to visualize nuclei, incubated for 24 h, washed with PBS, and mounted on glass slides with Prolong Gold mounting Medium (Invitrogen), adapted from Klimovich et al. (2020). All sections were analyzed using the laser scanning confocal microscope Olympus FV1200, visualized with the FV10-ASW software (Version 4.2, Olympus, Tokyo, Japan), and composed in Photoshop CS6.

### 2.9 Quantification and statistical analysis

Statistical analysis was conducted using two-way ANOVA, followed by Tukey’s *post hoc* test. All data are presented as the mean ± S.D. A *p*-value of < 0.05 was considered to indicate a significant difference. Statistical analysis was performed using GraphPad InStat3 (GraphPad, La Jolla, CA, United States) and StatView (version 5.0, SAS Institute Inc., Cary, NC, United States) for Windows. The significance level was set at *p* < 0.05 (*), *p* < 0.01 (**), and *p* < 0.001 (***). The correlations between LINX and TrkA levels in DRG, SC, and Cx were determined by using the Pearson’s correlation coefficient. Again, the significance level was set at a *p*-value of < 0.05.

## 3 Results

### 3.1 Investigation of LINX expression in the adult nervous system

#### 3.1.1 LINX expression in adult mouse and rat brain

The protein lysate extracted from the adult Rat Cx, 6-month and P1 mouse brain (Br 6 Ms. and Br P1, respectively) was analyzed by WB, as well as the Spinal Cord (SC) and Olfactory Bulb (OB) from 6-month CD1 mice, using the anti-LINX antibodies of previous studies (Abudureyimu et al., 2018). As shown in Figure 1A, a band corresponding to LINX molecular weight (MW 75–100 kDa) was clearly detected by the Abnova antibody, as also found by Abudureyimu et al. (2018), in all the run samples. A slightly weaker band is seen only in SC and OB. Figure 1B shows that no signals were detectable by using the antibody from R&D.

LINX expression in adult rat SC, DRG, and Cx was further analyzed by WB using the different commercial antibodies described in the Materials and Methods section. A band approximately 80 kDa, which is described as LINX protein (predicted MW is 79 kDa), was detectable in the SC and Cx samples using Abnova, Sigma-Aldrich, and Biorbyt anti-LINX antibodies, while ProSci did not detect any bands under our experimental conditions. Only the Abnova antibody showed a signal in all three tissues analyzed, including DRG (Figure 2); thus, this antibody was used in the *in vitro* experiments.

#### 3.1.2 LINX and TrkA interaction

Immunoprecipitation (IP) analysis was performed using rabbit anti-TrkA-specific antibody or rabbit anti-LINX antibody on cortical extracts from LPS-treated (LPS) or age-matched untreated control (CTRL) rats. We observed LINX binding to TrkA in the Cx under both physiological and inflammatory conditions, by both probing LINX on the eluate from TrkA immunoprecipitation (Figure 3A) and probing TrkA in the eluate from LINX immunoprecipitation (Figure 3B). Furthermore, by analyzing cortical samples from rats challenged with LPS, we found that the LINX-TrkA interaction is maintained by inflammatory conditions, as suggested by the TrkA bound to LINX upon LPS incubation (Figure 3A) As a confirmation that LINX and TrkA immunoprecipitation were specific and effective, LINX probing by anti-LINX-HRP antibody and TrkA probing by anti-TrkA-HRP antibody, respectively, confirmed enrichment of the IP proteins in the eluate (IP) and absent in the Flowthrough (Ft) (see Supplementary Figures S3A,B). The expression levels of LINX and TrkA in the cortex of CTR and LPS rats are shown in Supplementary Figure S3C.

### 3.2 Studies on organotypic culture of adult DRG

#### 3.2.1 Effects of NGF in LPS-challenged DRG in culture

The expression of axon growth markers such as GAP43 and TrkA was analyzed in CTRL and LPS-treated DRG, with or without NGF treatment. As shown in Figure 4A, a barely noticeable basal GAP43 positivity was found in CTRL DRG, while an increase of GAP43-expressing neurites (DRG outgrowth) was observable following NGF addition (Figure 4B). In LPS DRG, some GAP43 fibers were visible (Figure 4C), and an increase of GAP43/TrkA positive neurites outgrowth was still observable following NGF treatment (Figure 4D), indicating that LPS DRG are still able to respond to the neurotrophins.

**Figure 4:**
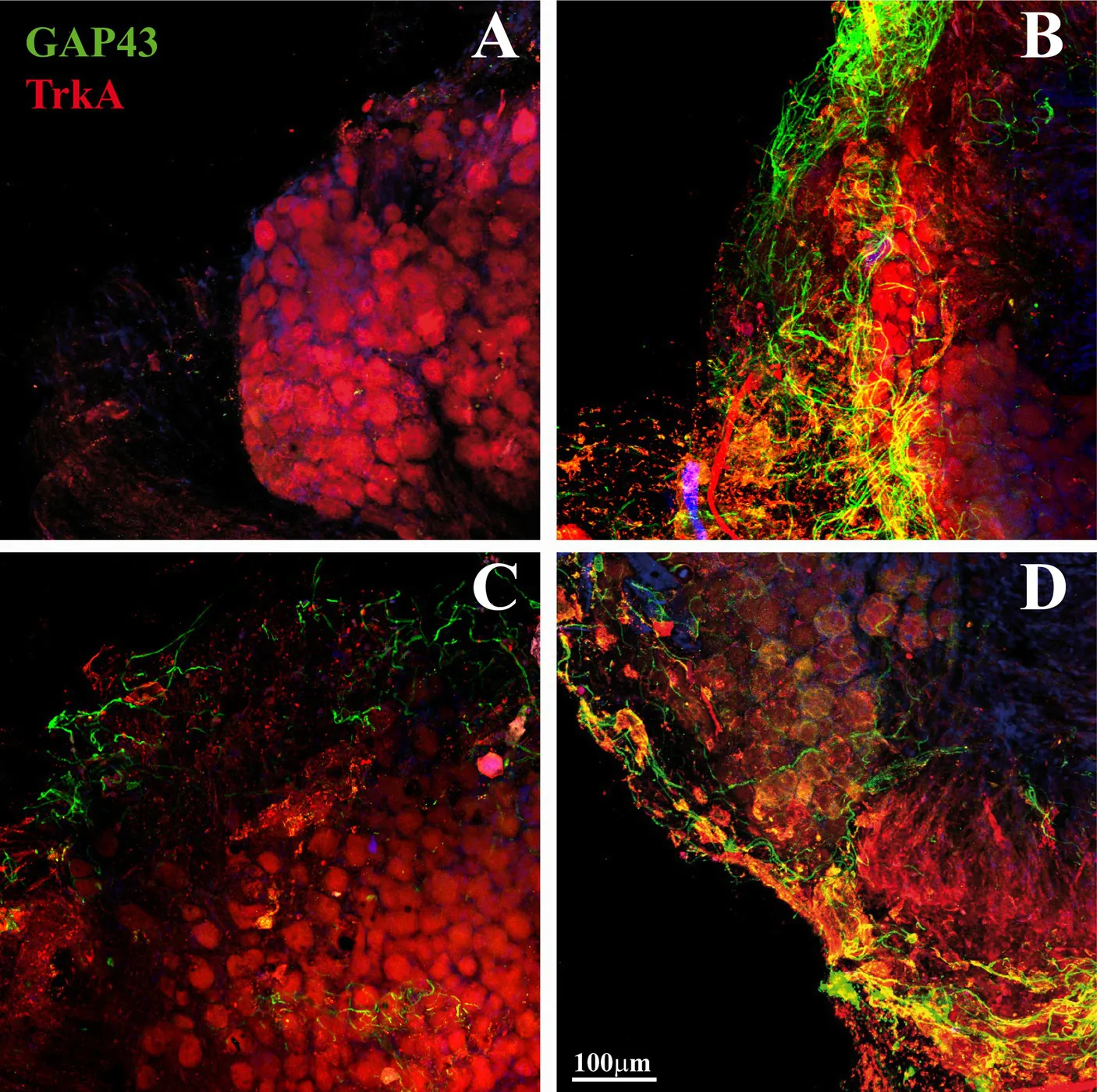
**(A–D)** Immunofluorescence of TrkA and GAP-43 on CTRL adult rat DRG or upon LPS challenge, with or without NGF treatment. **(A)** Immunofluorescence using GAP-43 (green), TrkA (red) antibodies, and DAPI on untreated DRG organotypic culture (CTRL), **(B)** on DRG organotypic culture treated with NGF (+NGF), **(C)** on LPS DRG organotypic culture (LPS), and **(D)** on LPS DRG organotypic culture treated with NGF (LPS + NGF).

#### 3.2.2 Effects of anti-LINX on LPS-challenged DRG organ cultures

The effects of anti-LINX on the expression of TrkA activation (pTrkA) and GAP43 in cultures of CTRL and LPS DRG were investigated by using the PathScan assay and WB analysis, respectively. As shown in Figure 5, both phosphorylated TrkA form levels increased in the LPS group compared to the CTRL group (Tyr490: **p < 0.01; Tyr 674/675: *p < 0.05). In the same graph, it can be observed that anti-LINX treatment in the LPS group (LPS + anti-LINX) resulted in a significant decrease of TrkA phosphorylation on both Tyr490 and Tyr674/675 residues when compared to the other groups (vs. LPS: **p < 0.01; vs. CTRL Tyr674/675: **p < 0.01, Figure 5A). The anti-LINX administration recovered the LPS-induced decrease of GAP43 expression, raising the protein level slightly above that of the untreated DRG group (CTRL vs. LPS: **p < 0.01, LPS vs. LPS + anti-LINX: ***p < 0.001; Figure 5B).

**Figure 5:**
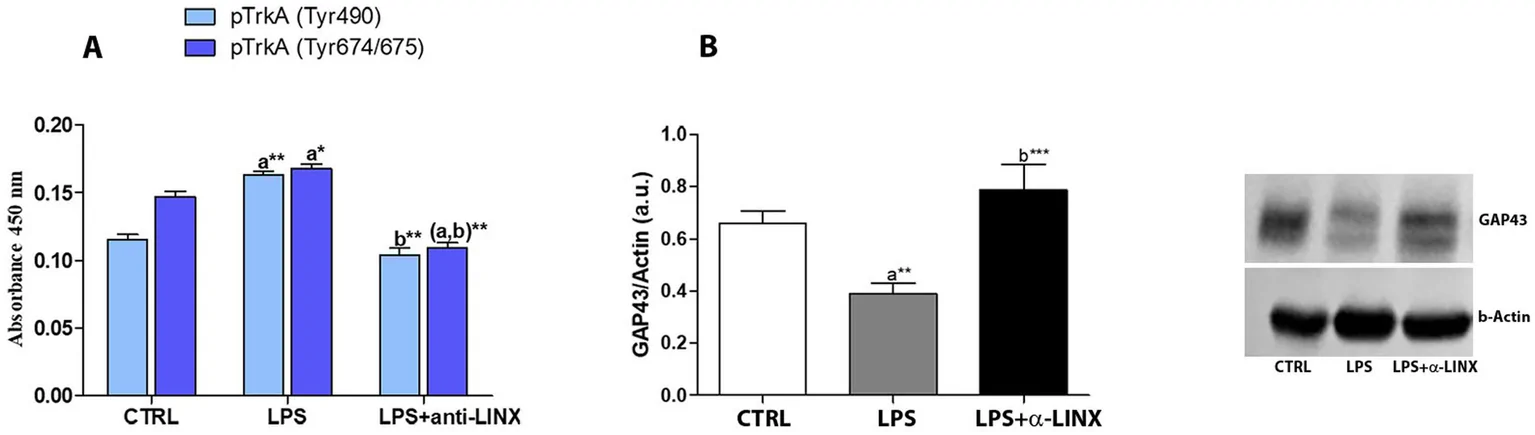
Effects of LPS and anti-LINX on DRG organ cultures **(A,B)**. **(A)** Phospho-TrkA PathScan assay performed on protein lysates from untreated (CTRL) and LPS DRG organotypic culture protein lysates (LPS). **(B)** Western blot analysis performed on protein lysates from CTRL and LPS DRG organotypic culture using anti-GAP-43 antibody. Data are expressed as the mean optical density (arbitrary units, a.u.) of three technical replicate wells of DRG pool as described in the M&M section. Statistical significance was calculated using two-way analysis of variance (ANOVA) for repeated measures followed by Tukey’s *post hoc* test, indicated with ***p < 0.001, **p < 0.01, and *p < 0.05. a vs. CTRL; b vs. LPS. β-actin was used as a loading control.

#### 3.2.3 Combined effects of NGF and anti-LINX in LPS DRG

The effects of NGF and anti-LINX on phosphorylated TrkA (pTrkA) on Tyr490 and Tyr674/675, or on GAP43, were studied in LPS DRG (Figures 6A,B). When NGF was added to DRG from the LPS group (LPS + NGF), a slight decrease in pTrkA on Tyr490 was observed (*p < 0.05; Figure 6A), while a significant upregulation of TrkA phosphorylation at Tyr674/675 sites was found (**p < 0.01; Figure 6A) when compared to the NGF-untreated LPS group (LPS). No changes in GAP43 were detected in the LPS + NGF group compared to LPS (Figure 6B). In Figure 6A, the level of pTrkA on both Tyr490 and Tyr674/675 phosphorylation sites was significantly decreased in the LPS + NGF + anti-LINX group when compared to LPS (vs. Tyr490: ***p < 0.001; vs. Tyr674/675 **p < 0.01); and LPS + NGF group (vs. Tyr490: **p < 0.01; vs. Tyr674/675 ***p < 0.001). At variance with LPS DRG with or without NGF treatment, a reduction of GAP43 was found in LPS DRG following treatment with NGF + anti-LINX (***p < 0.001; Figure 6B).

**Figure 6:**
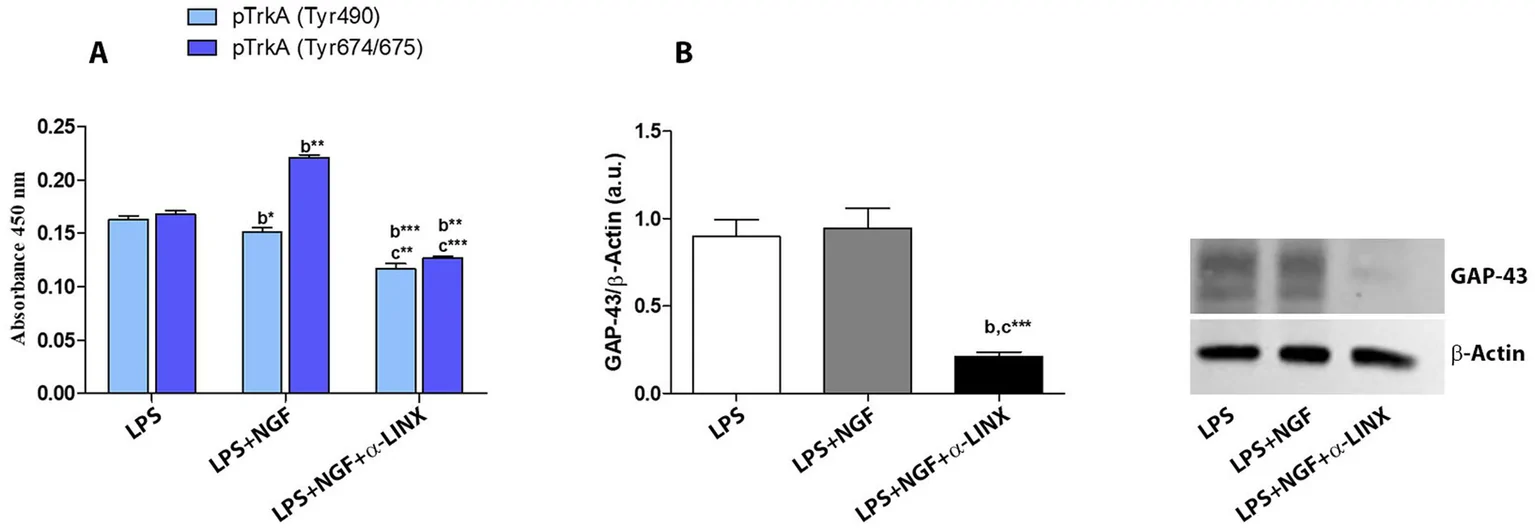
**(A,B)** Effects of NGF and anti-LINX in LPS DRG. **(A)** Phospho-TrkA PathScan assay performed on protein lysates from LPS DRG organotypic culture (LPS), treated with NGF (LPS + NGF), or treated with NGF + anti-LINX (LPS + NGF + anti-LINX). **(B)** WB analysis was performed on protein lysates from LPS- and LPS DRG-treated groups using anti-GAP-43 antibody. β-actin expression was used to normalize sample variability. For each treatment group, three technical replicate wells were prepared from the same DRG pool as described in the M&M section. Data are expressed as mean optical density in arbitrary units (a.u) and are given as mean SD. Statistical significance was calculated using two-way analysis of variance (ANOVA) for repeated measures followed by Tukey’s *post hoc* test, indicated as ***p < 0.001, **p < 0.01, and *p < 0.05. b vs. LPS; c vs. LPS + NGF.

#### 3.2.4 Effects of TrkA inhibitor in LPS-treated DRG

The effects of TrkA inhibitor in LPS DRG treated with or without anti-LINX are shown in Figures 7A,B. Anti-LINX or TrkA inhibitor administration in LPS DRG strongly decreases the TrkA phosphorylation at both sites (***p < 0.001) when compared to the LPS group. A significant increase in the levels of TrkA Tyr674/675 phosphorylation was found in the LPS + TrkA inhibitor group when compared to LPS + anti-LINX (***p < 0.001; Figure 7A). The combined treatment of anti-LINX and TrkA inhibitor (LPS + anti-LINX+TrkA inhibitor group) resulted in an increase of TrkA phosphorylated sites when compared to the single treatments (vs. anti-LINX: Tyr490 **p < 0.01; Tyr674/675 ***p < 0.001; vs. TrkA Inhibitor: Tyr674/675 ***p < 0.001). Confirming the set of experiments reported in paragraph 3.2.2, anti-LINX induces a significant upregulation of GAP43 in LPS DRG when compared to CTRL and LPS groups (**p < 0.01; ***p < 0.001, respectively, Figure 8B). Reduced expression levels of GAP43 were found in LPS DRG following treatment with TrkA inhibitor vs. CTRL (**p < 0.01, Figure 7B). The co-administration of anti-LINX and TrkA inhibitor in LPS DRG resulted in significantly higher GAP43 expression levels when compared to all the other experimental groups (***p < 0.001; Figure 7B).

**Figure 7:**
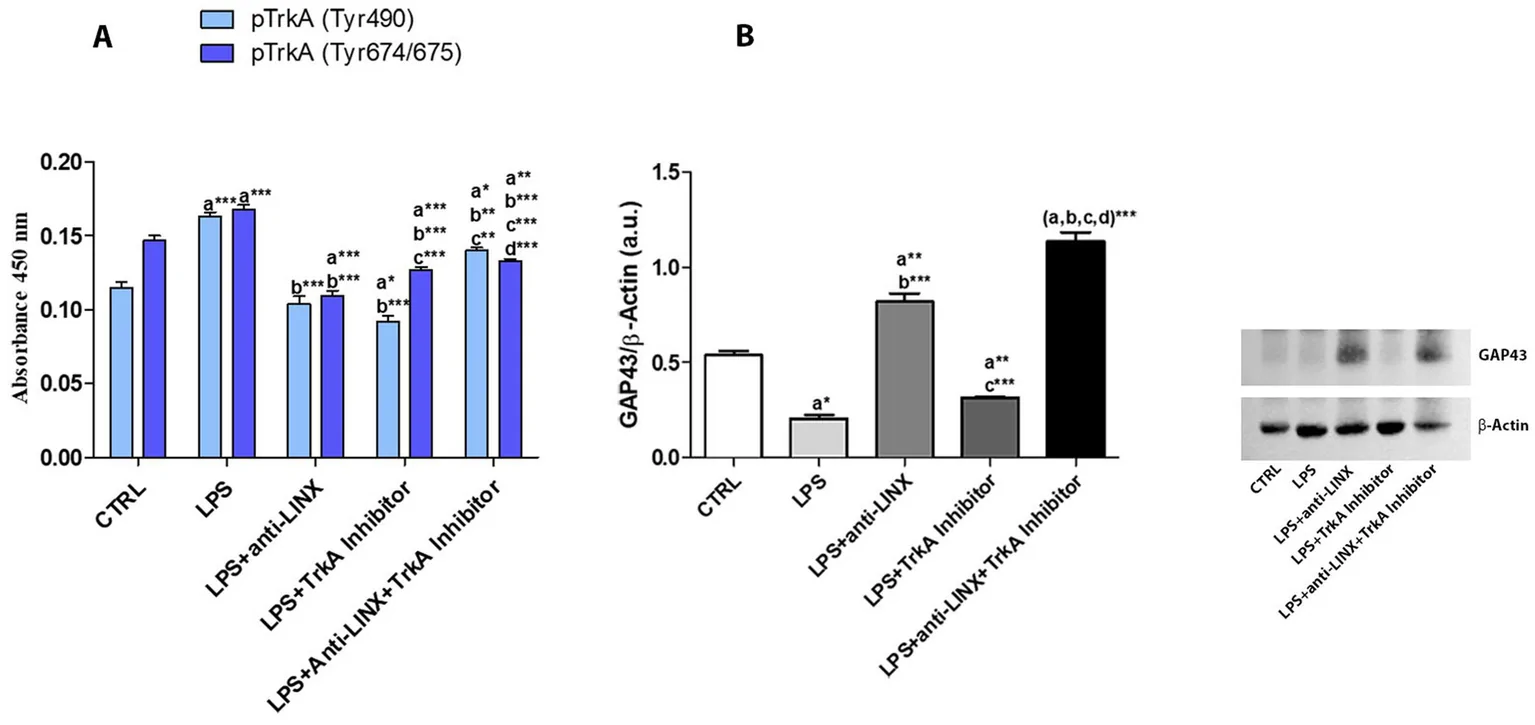
Effects of TrkA inhibitor and anti-LINX on the phosphorylation of TrkA **(A)** and the expression of GAP43 **(B)**. **(A,B)** Phospho-TrkA PathScan assay performed on protein lysates from LPS DRG organotypic culture treated with *α*-LINX (LPS + α-LINX) or treated with TrkA inhibitor (LPS + TrkA inhibitor) or with co-administration of α-LINX and TrkA inhibitor (LPS + α-LINX+TrkA inhibitor). **(B)** Western blot analysis was performed on protein lysates from LPS- and LPS DRG-treated groups using anti-GAP-43 antibody. β-actin expression was used to normalize sample variability. Three technical replicate wells for each treatment group were prepared from the same DRG pool as described in the M&M section. Data are expressed as mean optical density in arbitrary units (a.u.). Statistical significance was calculated using two-way analysis of variance (ANOVA) for repeated measures, followed by Tukey’s post hoc test, indicated as ***p < 0.001, **p < 0.01, *p < 0.05. A vs. CTRL; B vs. LPS; C vs. LPS + α-LINX; D vs. LPS + TrkA inhibitor.

**Figure 8:**
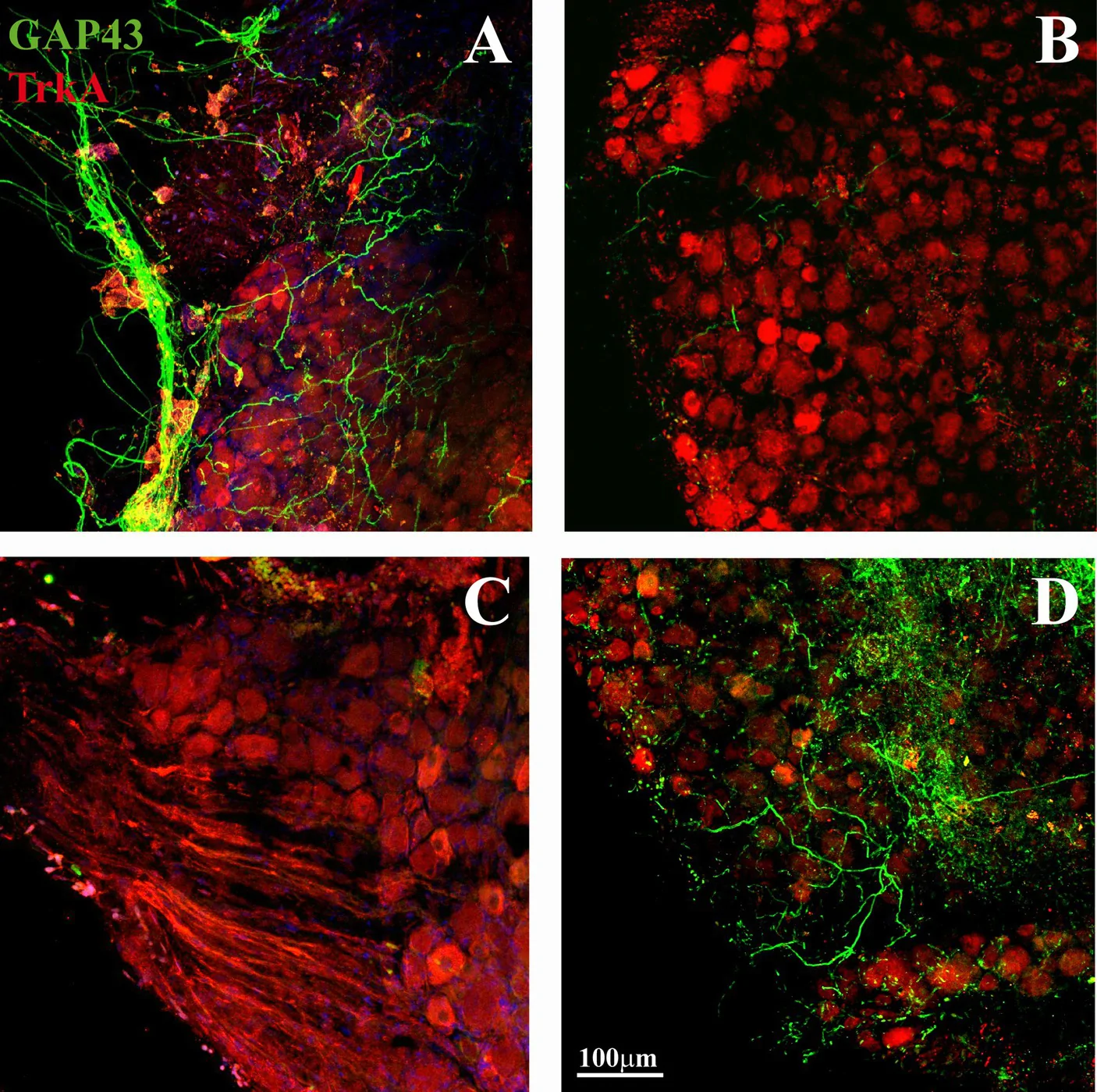
**(A-D)** Representative immunofluorescent staining of TrkA and GAP-43 on LPS-challenged adult rat DRG with anti-LINX or anti-TrkA (GW441756) inhibitors. **(A)** IF using GAP-43 (green), TrkA (red) antibodies, and DAPI on LPS-challenged adult rat DRG with anti-LINX inhibitor (LPS + anti-LINX), **(B)** or with NGF treatment (LPS + NGF + anti-LINX), **(C)** or LPS-challenged adult rat DRG with anti-NGF inhibitor (LPS + GW441756), alone **(D)** or with anti-LINX antibody (LPS + anti-LINX+GW441756).

#### 3.2.5 IF for TrkA and GAP-43 in LPS DRG treated with anti-LINX antibody

IF was performed on CTRL and LPS DRG organotypic culture treated with NGF, anti-LINX, or TrkA inhibitor using antibodies against GAP43 (green) and TrkA (red), and 4′,6-diamidino-2-phenylindole (DAPI) to counterstain the nuclei (blue) (Figure 8). The confocal observations confirmed the molecular analysis, showing that anti-LINX stimulates GAP43 expression in LPS (Figure 8A) and alters the NGF effects (Figure 8B). A stable TrkA positivity and undetectable GAP43 staining was observable in LPS DRG following TrkA inhibition (Figure 8C), while GAP43-stained neurite persists in LPS DRG treated with anti-LINX and TrkA inhibitor (Figure 8D).

## 4 Discussion

The LINX was found to be expressed in developing mouse brain (Br), spinal cord (SC), and dorsal root ganglia (DRG) (Abudureyimu et al., 2018; Mandai et al., 2009; Mandai et al., 2014; Panza et al., 2015), and identified as a component associated with TrkA receptor signaling during neuronal development, which facilitates/contributes to axonal growth, guidance, and branching. Accordingly, a physical interaction between LINX and TrkA has previously been demonstrated using embryonic DRG (Abudureyimu et al., 2018). Neither the presence of LINX nor its interaction with TrkA in the adult rat nervous system nor the role of LINX in injured adult neurons were investigated previously. These questions were addressed in the present study by examining LINX expression in the adult Cx, SC, and DRG, and the effects of systemic inflammation on the LINX/TrkA association in the adult Cx. Organotypic cultures of lumbar DRG from adult rats receiving LPS were also used to explore LINX contribution to axon growth in control and inflammatory conditions.

Using the same antibodies reported by Abudureyimu et al. (2018), we were able to confirm the expression of LINX protein in total brain tissues, such as Cx, and from postnatal day 1 (P1) mice, as well as in Cx from young adult (6 months old) CD1 male mice, and adult rats. Commercial antibodies obtained with different immunogenic peptides were used and compared to analyze the presence of LINX in adult rat SC, DRG, and Cx by WB. No detectable signals were obtained using ProSci and R&D antibodies, while the Abnova, Sigma, and Biorbyt antibodies were able to recognize LINX in Cx and SC. However, only the Abnova antibody shows a band approximately 80 kDa in DRG. To avoid an incorrect estimation of LINX expression, the Abnova antibody was selected to further investigate LINX in adult Cx and characterize LINX action on axonal growth using the DRG LPS model.

As shown in Figure 3, the immunoprecipitation study demonstrated that LINX is associated with TrkA expression in both CTRL and LPS adult Cx, and that systemic LPS might acutely affect the expression of TrkA (Supplementary Figure S3) but not LINX in adult Cx. These findings suggest that the two proteins might be differentially regulated during the activation of the inflammatory cascade, but that the LINX/TrkA interaction is maintained in the LPS-induced cortical injury condition.

To further investigate the role of LINX in injured neurons, and since no anti-LINX Fc antibody or selective LINX inhibitor is available, the Abnova anti-LINX antibody was used as a tool to interfere with the NGF/TrkA-mediated response treatment in the LPS DRG model. As mentioned in the introduction, LPS has been shown to compromise brain/DRG–blood barrier (Reinhold and Rittner, 2020), increase paracellular permeability (Bein et al., 2017), and stimulate endocytic internalization and transient membrane destabilization (Zanoni et al., 2011), and in turn allow the aberrant penetration of macromolecules inside the organs. Thus, in our study, the challenge with LPS, besides representing a model largely used in the neurotrophin neuroscience field (Qin et al., 2007), was used also to foster the anti-LINX antibody permeability in organotypic DRG cultures. As shown in Figure 4, we found that NGF stimulates GAP43 and TrkA, confirming that, in our experimental conditions, the LPS DRG maintains its ability to respond to NGF by stimulating axon growth markers (Neto et al., 2017; Aoki et al., 1976).

Consistent with the idea that LINX expression in DRG might be associated with the NGF/TrkA pathway, treatment with anti-LINX antibody was found to alter LPS-stimulated TrkA activation in LPS DRG when administered in combination with NGF (Figures 6A,B). A marked decrease in GAP43 was also observed when comparing NGF and NGF + anti-LINX treatments, supporting the involvement of LINX in the NGF-mediated action on cultured/injured adult DRG, as observed during development (Mandai et al., 2009).

Nonetheless, our biomolecular analysis and confocal studies show an increase in GAP43 when anti-LINX is administered alone or in combination with a TrkA inhibitor. The effects of anti-LINX on the axon growth marker do not appear associated with TrkA activation, as supported by the findings that the PathScan assay, which is specific for TrkA and TrkA phosphorylation sites, and since the WB of GAP43 (Figure 7) were run using the protein samples from the same LPS DRG pools (see experimental protocol description in Materials and Methods). In addition, as shown in Figure 8, anti-LINX administration results in high GAP43 expression in LPS DRG, which is only partially suppressed by treatment with a TrkA inhibitor, further indicating that anti-LINX action on the axon growth marker might not be exclusively associated with TrkA activation in adults.

As a member of LRR, LINX is able to interact with all Trk receptors, as well as RET. Thus, it is possible that these receptors might be involved in the mechanism by which anti-LINX stimulates axon growth in our model of LPS DRG. In fact, the brain-derived neurotrophic factor (BDNF) is released by DRG in culture following NGF or tumor necrosis factor-α (TNF-α) (Lin et al., 2011), and BDNF/TrkB stimulates regeneration in injured ascending DRG neurons (Song et al., 2008). The neutrophin-3 (NT3) and TrkC are also upregulated in injured DRG, and NT3 derived from satellite glia cells contributes to sympathetic sprouting following peripheral nerve injury (Tender et al., 2011; Zhou et al., 2008).

In this context, it is worth noting that in lumbar DRG RET is co-expressed with all the Trks, while a small percentage of neurons express RET and TrkA but not TrkB or TrkC (Ernsberger, 2008). Kashiba et al. (1998) found that following axotomy, these neurons lose TrkA but not RET, representing a subpopulation of DRG neurons that remains responsible in the absence of NGF and/or reduced TrkA. As shown in Figure 8, a low co-expression of GAP43 and TrkA is still observable following combined treatment with a TrkA inhibitor, suggesting that TrkA/RET neurons might respond to the anti-LINX treatment in the presence of the TrkA inhibitor. Experiments are currently ongoing in our laboratory to characterize the cell type-specific expression of LINX in adult DRG, and the effects of anti-LINX on the expression levels of neurotrophins and glial derived neurotrophic factor (GDNF) and their receptors in the LPS DRG models.

To summarize, our study confirms that LINX is expressed in the adult central nervous system and demonstrates that LINX expression in adult Cx is not affected but LINX/TrkA interaction is maintained following LPS-induced systemic inflammation. Moreover, our data indicate that antibody-mediated perturbation of endogenous LINX is associated with modulation of axonal growth markers in adult LPS DRG cultures, in serum-free conditions and/or in the presence of a TrkA inhibitor.

As mentioned before, the few studies on LINX and the lack of availability of monoclonal or Fc antibodies, as well as synthetic LINX inhibitors, led us to select a polyclonal antibody as a tool to interfere with endogenous LINX in organotypic DRG cultures. The major limitations of this approach are that the Abnova antibody was not validated as neutralizing by the vendor, and that polyclonal antibodies might react with different epitopes, resulting in less specificity and potentially activating structurally similar molecules. In our study, the use of isotypic IgG as a control and the findings that LPS DRG respond to NGF under our experimental conditions help verify the functionality of our *in vitro* model. Furthermore, the observation that anti-LINX modulates TrkA activation and alters the NGF-induced increase in GAP43 expression in LPS-treated DRG cultures supports a role for endogenous LINX in regulating NGF/TrkA-dependent signaling that underlies axonal growth ex vivo. Thus, although the effects of anti-LINX on the regulation of GAP43 in injured DRG and the mechanisms that might occur without the contribution of TrkA need to be further characterized, the data presented in our study offer a new insight for future investigations on the role of LINX in the regulation of neuronal functions in adults.

## Data Availability

The raw data supporting the conclusions of this article will be made available by the authors, without undue reservation.
