# Supplementary material for: Beyond development: LINX/Islr2 expression and TrkA interaction in the adult nervous system under neuroinflammatory challenge
Source: Front Mol Neurosci. 2026 Sep 23;19:1863350. doi: 10.3389/fnmol.2026.1863350 (PMC13642797; doi:10.3389/fnmol.2026.1863350)
Supplement: Supplementary file 1 [file Data_sheet_1.pdf]

Supplementary materials

Original Images for Blots and Gels Requirements

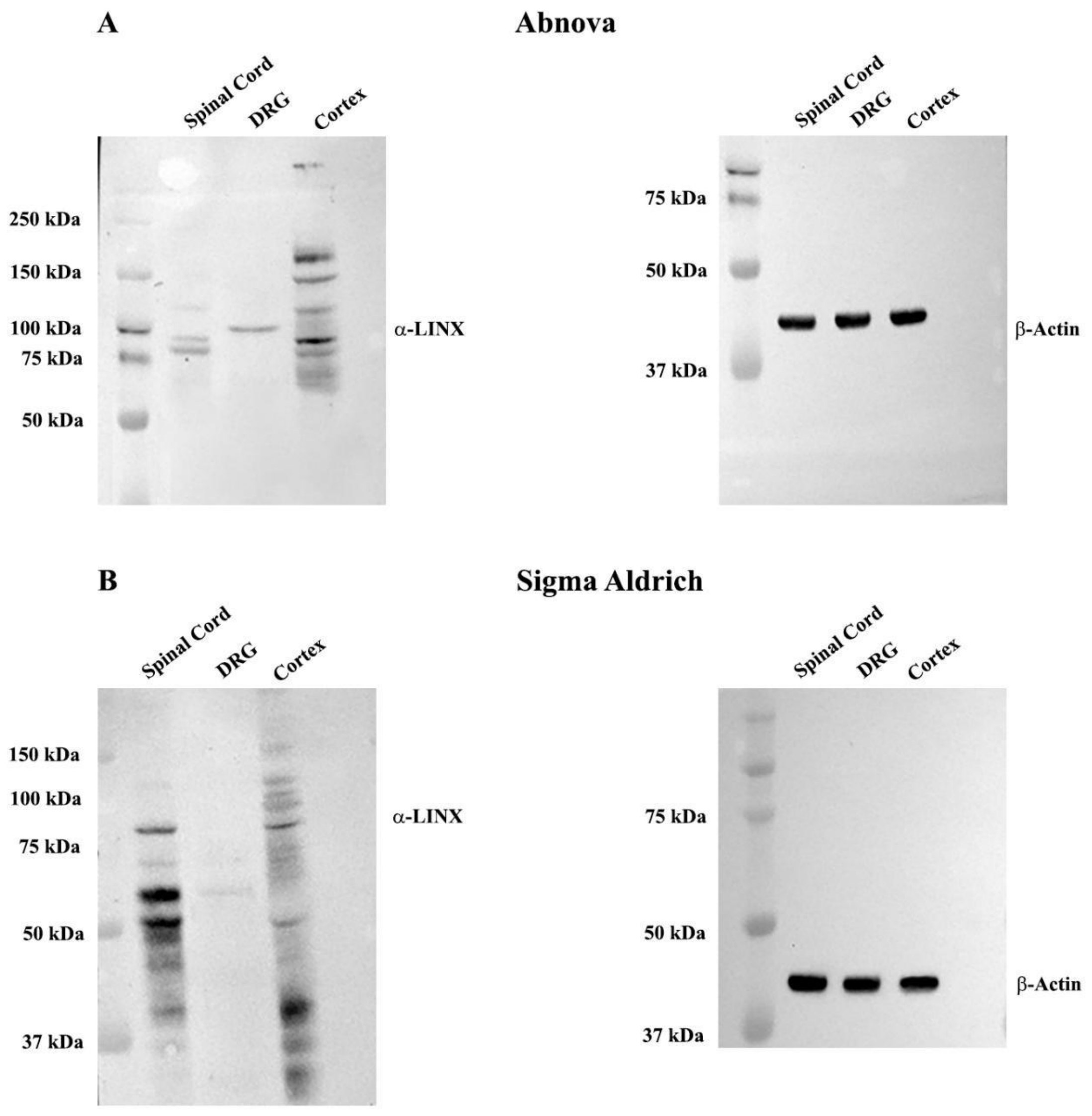

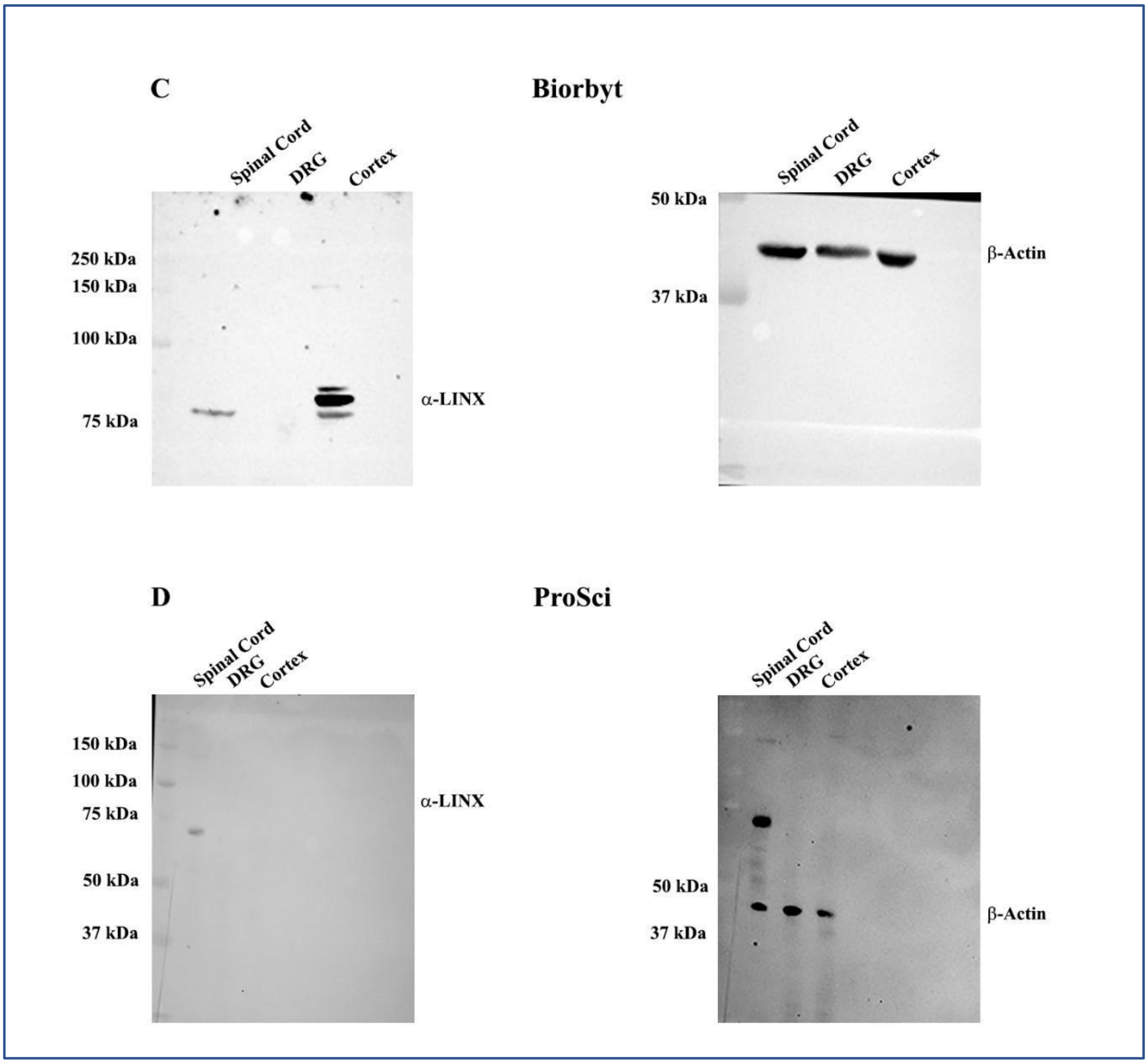

Figure S2. Original image for blots corresponding to the **Figure 2**

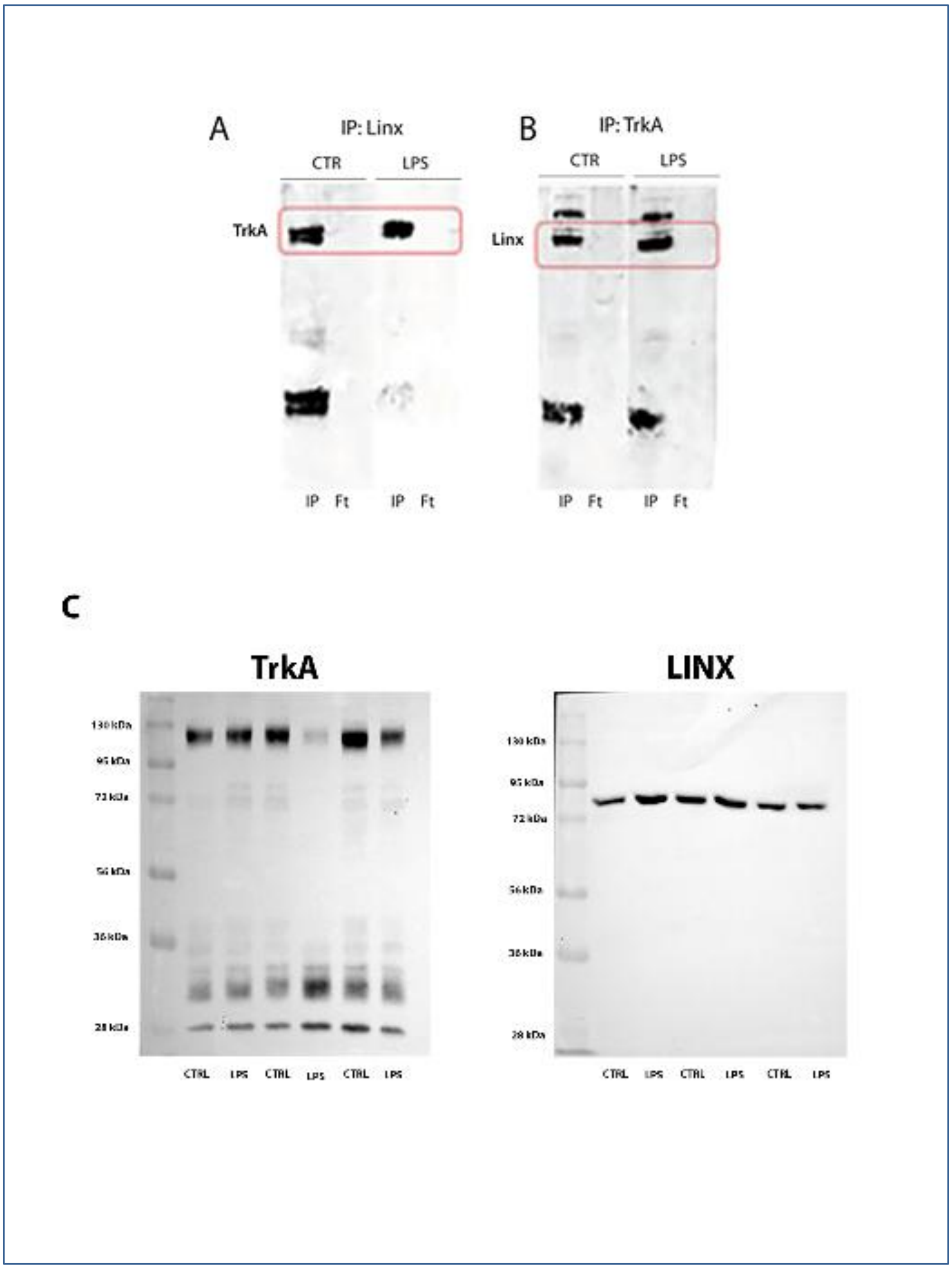

Figure S3. Original image for blots corresponding to **Figure 3.**

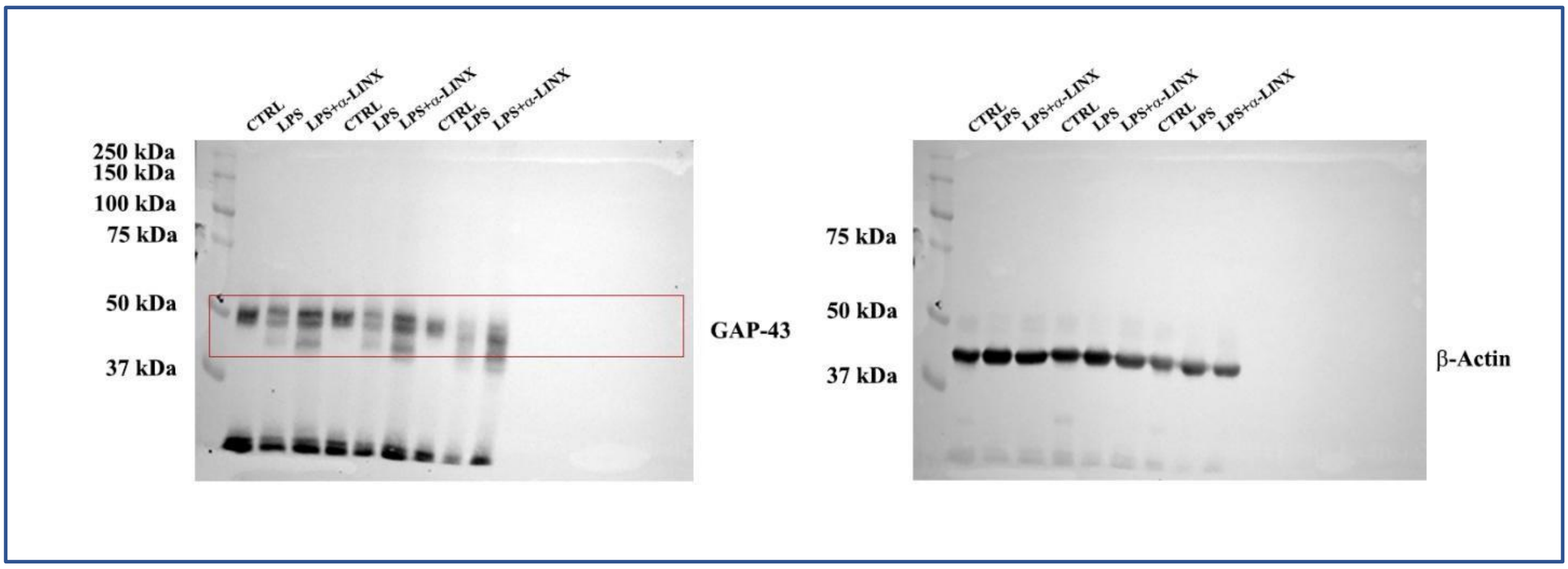

Figure S5. Original image for blots corresponding to DRG in the **Figure 5.**

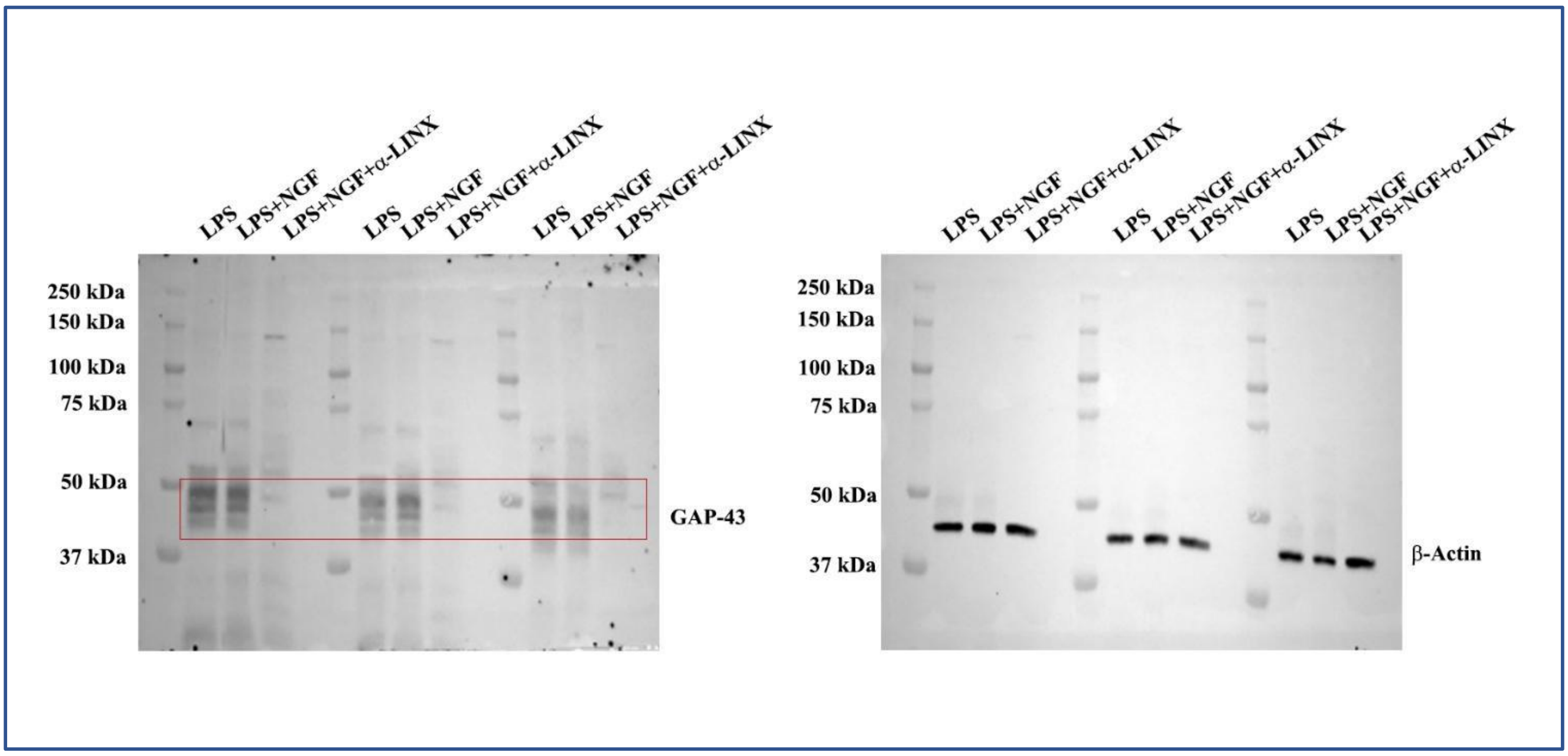

Figure S6. Original image for blots corresponding to DRG in the **Figure 6.**

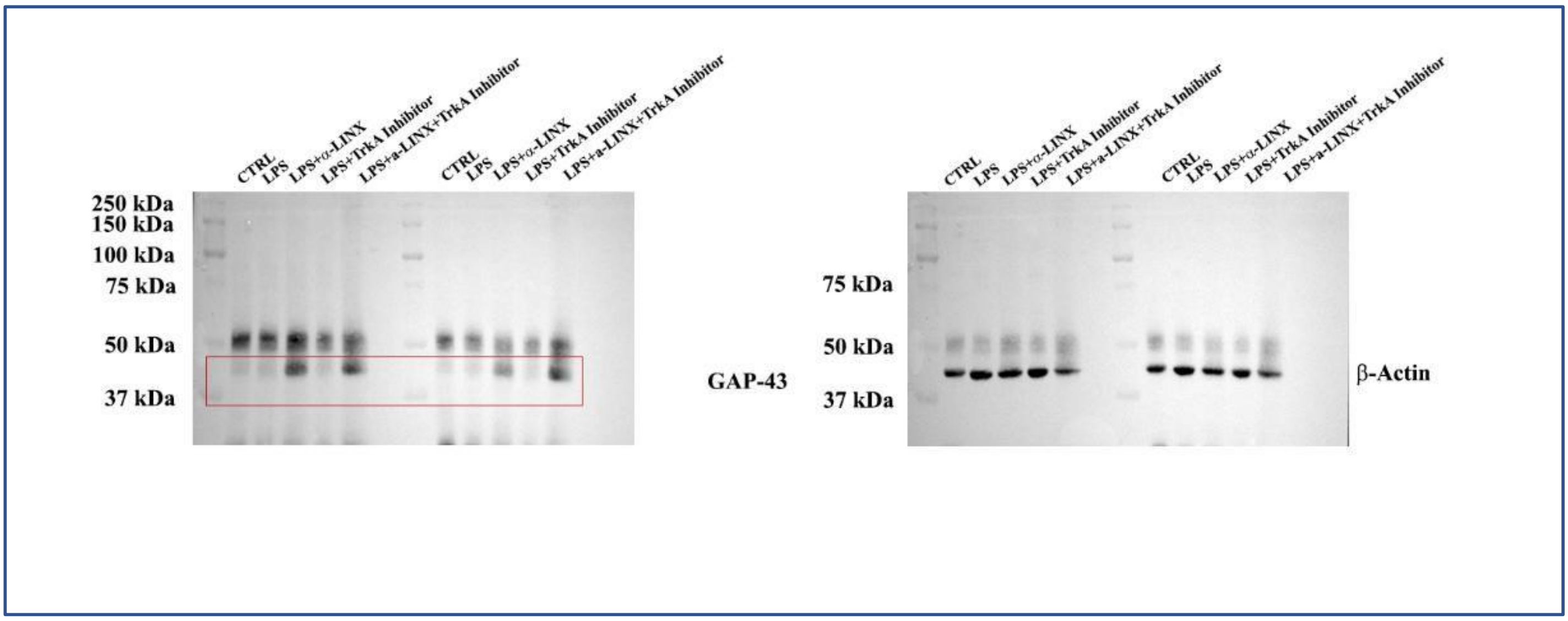

Figure S7. Original image for blots corresponding to DRG in the **<u>Figure 7.</u>**
